# Photography, medicine and corporeality: the device of the image in the forms of discursive apprehension of the disease

**DOI:** 10.1590/S0104-59702024000100020

**Published:** 2024-05-17

**Authors:** Hilderman Cardona Rodas

**Affiliations:** i Profesor investigador, Facultad de Ciencias Sociales y Humanas/Universidad de Medellín. Medellín – Antioquia – Colombia hcardona@udemedellin.edu.co

**Keywords:** Body, Disease, Photography, Medical imaging, Dermatology, Cuerpo, Enfermedad, Fotografía, Imagen médica, Dermatología

## Abstract

To study about and reflect on the disease is to highlight the ways of seeing and saying what can a body and its power to be affected before fingerprints or traces that degrade it. This article exposes epistemological research on social representations brackets (where register know doctor) disease from the registry of Clinical Dermatology in the second half of the 19th century. This is resorted to an analysis of medical photographs preserved in archives of Colombia and Spain taking as discursive forms of seeing and saying the disease who have disfiguring effects in the body.

## Resumen

Estudiar y reflexionar sobre la enfermedad es poner de relieve las formas de ver y decir acerca de lo que puede un cuerpo y su potencia de ser afectado ante las huellas o vestigios que lo degradan. Este artículo expone los soportes epistemológicos de una investigación sobre las representaciones sociales (en la que se inscribe el saber médico) de la enfermedad desde el registro de la dermatología clínica durante la segunda mitad del siglo XIX. Para esto, se recurrió a un análisis de fotografías médicas conservada en archivos de Colombia y España y como horizonte discursivo las formas de ver y decir la enfermedad que tiene efectos deformantes en el cuerpo.

**Venus Anadiomena**
Como de un ataúd verde, en hoja de lata,
con pelo engominado, moreno, y con carencias
muy mal disimuladas, de una añosa bañera
emerge, lento y burdo, un rostro de mujer.
El cuello sigue luego, craso y gris, y los hombros
huesudos, una espalda que duda en su salida
y, después, los riñones quieren alzar el vuelo:
bajo la piel, el sebo, a capas, como hojaldre.
El espinazo, rojo, y el conjunto presentan
un regusto espantoso, y se observa ante todo
detalles que es preciso analizar con lupa.
El lomo luce dos palabras: CLARA VENUS.
Un cuerpo que se agita y ofrece su monture
hermosa, con su úlcera, tenebrosa, en el ano
(Rimbaud, 1996).

Las monstruosidades físicas, que ya habían sido objeto de debate desde la Antigüedad, se constituyen entre los siglos XVII y XIX en un desafío a la normatividad taxonómica de las ciencias naturales (Foucault, 2001a). Al finalizar el siglo XIX, los modos de existencia corporal monstruosa o deforme otorgan materiales de reflexión clínica a la medicina de la época. Las descripciones de enfermedades, en tanto experiencias patológicas en los pacientes, son un lugar de enunciación del saber-poder médico y estaban acompañadas de imágenes (fotografías o dibujos) en un campo de inmanencia epistemológica en el ejercicio de la mirada clínica. El uso de las imágenes, incluida la fotografía médica, tuvo la función de exhibir lo diferente y lo alterado, visto como grotesco frente a un ideal de normalidad orgánica y psicológica.[1]

Las diferentes modalidades de la imagen, en la aprehensión del cuerpo enfermo, proyectan algo fundamental: estéticas del dolor y efectos de la enfermedad sobre los cuerpos. La imagen como representación, en cuanto capta, cautiva y es captada, hace de la iconografía médica un campo de expresión de fuerzas, creencias y un acontecimiento en la experiencia corporal de la existencia humana. Dibujos, pinturas o fotografías en los que se reportan enfermedades que se revelan en la piel, como superficies patológicas que muestran un vínculo semiológico de una doble grafía, es decir, palabra e imagen se relacionan ante la mirada de un cuerpo que expone la pluralidad de las formas de la vida.

> El enfermo, interrogado, da una lectura particular de sus malestares; evoca lo que para él es determinante en su estado. Un saber profano formaliza su experiencia, se explica el origen de la enfermedad que le afecta, su razón de ser, se inventa trucos que le permiten aliviar su desdicha. Pero la medicina no lo oye, porque participa de una cultura sabia, propia de un restringido grupo de facultativos formados para manipularla. El paciente está excluido de estas prácticas y conocimientos; el cuerpo al que alude está habitado por los movimientos y las imágenes de su vida cotidiana y de sus relaciones con los demás, sobre todo en su trabajo. Para el médico, el paciente está unido a un cuerpo abstracto, impregnado de una biología cuyas turbulencias se esfuerza por descubrir (Le Breton, 1999, p.146).

La relación que el médico estableció con el cuerpo enfermo pone de manifiesto una percepción del dolor y de los síntomas que el paciente experimenta ante la enfermedad, lo cual permitió delimitar categorías nosológicas que podrían ser conectadas a terapéuticas específicas para mitigar el dolor del paciente. "El médico tiene acceso a los contenidos corporales del enfermo (al menos en la primera etapa) a través de una palabra y una queja que pueden dar prioridad a percepciones para él triviales, y dejando en la sombra indicios mucho más reveladores" (Le Breton, 1999, p.146-147). Una de las formas de acercamiento de la práctica médica a las experiencias del dolor, aunque manteniéndose al margen, la constituye el uso de la imagen en la que se retratan cuerpos que exhiben las trazas de la enfermedad. Estas imágenes muestran la memoria de un cuerpo que es desmantelado desde su interior, como si la enfermedad y el dolor fueran formas de posesión que carcomen al individuo y su integridad corporal. En este horizonte de percepción médica opera una racionalidad objetiva desde el plano iconográfico, en el cual determinadas formas simbólicas de la subjetividad pasan a considerarse como expresiones peligrosas subjetivas al representarse con dibujos, pinturas o fotografías de enfermedades que comportan perturbaciones corporales en los pacientes. De acuerdo con Ortega (2010, p.113),

> los constructores de atlas anatómicos del siglo XVI al siglo XVIII consideraban que la objetividad se expresaba en la idea de fidelidad a la naturaleza, correspondiente a la producción y selección de imágenes consideradas típicas, características, ideales o medias. Para Goethe, por ejemplo, destilar lo típico (que remite a un arquetipo subyacente del cual puedan ser derivados los fenómenos individuales) de lo variable y de lo accidental no es considerado un deslizamiento hacia la subjetividad; es una forma de protección contra ella. Las imágenes típicas predominan en los atlas anatómicos hasta mediados del siglo XIX.

Estas imágenes típicas comienzan a cambiar a mediados del siglo XIX, cuando se fusionan dos formas de fidelidad a la naturaleza, una de representación de lo típico y otra de objetividad mecánica que presenta individuos reales y no ideales que, sin embargo, encarnan tipos ideales. Un ejemplo que pone en tensión una representación de lo típico (pintura) y una objetividad mecánica (fotografía) es el galope de los caballos:

> Millares de personas, durante siglos, han observado el galope de los caballos, han asistido a carreras y cacerías, han contemplado cuadros y grabados hípicos, con caballos en una carga de combate o al galope tras los perros. Ninguna de esas personas parece haberse dado cuenta de cómo se presenta realmente un caballo cuando corre. Pintores grandes y pequeños los han presentado siempre con las patas extendidas en el aire, como el gran pintor del siglo XIX Théodore Géricault en un famoso cuadro de las carreras de Epsom. Hace unos ciento veinte años, cuando la cámara fotográfica se perfeccionó lo suficiente como para poder tomar instantáneas de caballos en plena carrera, quedó demostrado que tanto los pintores como su público se habían equivocado por entero. Ningún caballo al galope se mueve del modo que nos parece tan 'natural', sino que mueve sus patas en tiempos distintos al levantarlas del suelo. Si reflexionamos un momento, nos daremos cuenta, que difícilmente podría ser de otro modo. Y sin embargo, cuando los pintores comenzaron a aplicar este nuevo descubrimiento, y pintaron caballos moviéndose como efectivamente lo hacen, todos se lamentaban de que sus cuadros mostraran un error (Gombrich, 1997, p.27-28; destaque en el original).

Siguiendo las líneas anteriores, en este artículo se examinan imágenes y discursos de la práctica médica en la segunda mitad del siglo XIX, teniendo como lugares de enunciación y producción discursiva a Colombia y España, desde donde se pone en obra el campo de saber de la dermatología clínica propio del proyecto biomédico de aquella época. En este sentido, al analizar imágenes y discursos apreciables en los archivos médicos de ambos países, se pone de manifiesto un pensamiento que se pliega en las formas de ver y decir la enfermedad que entraña transformaciones del cuerpo (lepra, sífilis, edemas, úlceras, escrófula, impétigo, dermatitis, lupus, entre otras), en las que un afuera implica un adentro observable, clasificable y analizable según el registro de la mirada clínica dermatológica que capta-describe al cuerpo enfermo. Aquí, el pliegue constituye un singular que avanza por variación, ya que se bifurca y se metamorfosea en una cadena significante en la que solo difieren las semejanzas y solo las diferencias se parecen.[2] Y es a través del uso de la imagen médica (dibujos, vaciados en cera o fotografías) como tal ejercicio de plegado del saber médico adquiere toda su capacidad de potencia en la configuración iconográfica de una semiótica de lo deforme en la asimilación de “lo que puede un cuerpo”[3] en estado de enfermedad. Así, lo prohibido e imposible se hace carne expresiva en la iconográfica médica, lo que ha tenido un espacio de reflexión en la historiografía latinoamericana,[4] por lo cual este artículo es un aporte a este campo de investigación en historia de la práctica médica y dermatología clínica ligada al uso del dispositivo de la imagen.

## Médico-fotógrafo-enfermo: un trípode iconográfico del cuerpo deformado

El desplazamiento hacia la objetividad mecánica ligada a los dispositivos tecnológicos del siglo XIX propicia una crítica al ideal de objetividad como fidelidad a un tipo, el cual se creía que tenía que ver con la presencia de un elemento subjetivo que entrañaba parcialidad. La imagen médica en el registro del dispositivo fotográfico consolida la idea de una objetividad mecánica en el momento en el que una mecanización se impone al arte en el cual reina la interpretación y el juicio individual, para contener la subjetividad. La fotografía médica,[5] difundida en los años cincuenta del siglo XIX, permitió, con la pretensión de un orden objetivo, corregir los errores subjetivos de las ilustraciones médicas, aunque se debe mencionar que ya la pose y toma fotográfica constituye una puesta en escena subjetiva en una escenografía o montaje.[6] Como lo indica Ortega (2010, p.115-116), “la imagen mecánica instaura un orden moral de auto restricción y comedimiento, pues en la elección entre la probidad moral y la representación precisa, el científico debía elegir la primera”. Médicos y fotógrafos asumirán la imagen fotográfica como una evidencia objetiva en un espacio discursivo positivista, pero, cuando se habla de fotografía, se remite a una intencionalidad realista asegurada por una mediación tecnológica que solo encuentra su eficacia en un trucaje óptico. El artefacto en sí no capta lo real, es la disposición de los elementos lo que permite que una escena fotografiada llegue a adquirir dimensión expositiva a través del ojo que organiza y capta en el registro de un saber específico, en nuestro caso, la dermatología clínica y el reconocimiento del cuerpo enfermo.

La imagen se convirtió en un instrumento para representar cuánto transgredía el horizonte de la normalidad anatomo-fisiológica. El dibujo, la pintura, la escultura en moldes de cera, y más tarde la fotografía médica, permitieron la configuración iconográfica de una semiología de lo monstruoso y lo deforme, según la distinción constante entre sano y patológico. Esta distinción fue estudiada por el filósofo e historiador de la medicina y la biología Georges Canguilhem (1952, 1971), quien reflexionó sobre esta desde el campo de las ciencias de la vida:

> Con la interrogación de los fenómenos patológicos (el problema de la especificidad de la enfermedad y del umbral que marca entre los seres vivos), el conocimiento de la vida pone al margen todo conocimiento referente a dominios físico-químicos y da estatuto propio a una pregunta por el viviente. Es precisamente por la posibilidad de la enfermedad, de la muerte, de la monstruosidad, del error o de la anomalía que se constituye una ciencia del viviente (Cardona Rodas, 2005, p.155).

Desde una perspectiva histórica, las ciencias de la vida tienen que ver con el problema filosófico del conocimiento, puesto que hablar de vida y muerte, en este registro, es desplegar preguntas que van más allá de cuestiones de física, al convocar asuntos de índole moral y política, no de cuestiones eminentemente científicas, según el campo o terreno que se analice, como es el caso del discurso médico. Por esta razón, y lo recuerda Canguilhem, Friedrich Nietzsche (1844-1900) valoriza lo patológico como homogéneo de lo normal, para con esto ver en los estados mórbidos un cristal de aumento de los procesos de la vida que resultan invisibles en el estado normal.

El cristal de aumento mencionado por Nietzsche se refleja en aquellos cuerpos que son retratados y que capturan lo otro, con el objeto de religar aquello que es visto como imposible con lo no permitido. La imagen (dibujo, retrato, molde o fotografía) registra el acontecimiento mórbido para codificar y hacer comprensible, en el registro, una política de la exhibición, el estereotipo del otro, que es imagen inversa de sí mismo. En esta medida, es posible trazar una reflexión sobre la dimensión social del cuerpo, para tener en cuenta la relación problemática entre lenguaje y enfermedad que se configura en la clínica, durante la segunda mitad del siglo XIX, según el horizonte del discurso de la dermatología en España y Colombia, espacios de producción discursiva desde donde se examinó el ejercicio de la mirada clínica dermatológica inaugurada por los trabajos de Jean-Louis Alibert (1768-1837) y continuada por Thomas Bateman (1821-1861), Jonathan Hutchinson (1828-1913) y Ernst Bazin (1807-1878), entre otros en Europa.[7] De acuerdo con los puntos anteriores, se pretende analizar la configuración del conocimiento médico en torno al grafema que hace de la escritura y la iconografía un dispositivo de comprensión ideográfica de la enfermedad deformante.

> La re-presentación narrativa e iconológica de la enfermedad deformante y de la monstruosidad, constituye un instrumento de conocimiento médico, en un puro grafismo de lo patológico que le da eficacia teórica al ejercicio de la mirada clínica. El ojo capta una equivalencia entre la voz de la enfermedad – que perturba al cuestionar una estructura morfológica normal – y la mano que graba en el cuerpo signos de aquello que es considerado desviación o desproporción en tanto deformidad, anormalidad o monstruosidad (Cardona Rodas, 2005, p.165).

En este dispositivo de la mirada clínica, en el que el cuerpo, bajo los rigores de la enfermedad, presenta una dimensión plástica, el uso de la imagen cobra un valor de captura y representación de lo patológico al darle un valor pedagógico y material de visibilidad de lo que es clasificado y concebido como enfermedad. Así, es apreciable cómo las descripciones clínicas y las representaciones iconográficas del cuerpo enfermo encuentran su soporte en las manifestaciones patológicas en una piel descubierta (Dagognet, 1993), que deja ver lo que puede un cuerpo como lenguaje representado. En esta perspectiva, dibujo, fotografía y observación clínica se reúnen en un espacio de figuración y representación ideográfica de lo que es visto como enfermedad. En el análisis de casos clínicos, caracterizaciones de enfermedades de la piel, además de las imágenes consignadas en tratados, álbumes, tesis y estudios comparativos, son apreciables las experiencias sensibles de lo patológico escenificadas en el cuerpo del paciente, las cuales son transmitidas por un discurso médico especializado que proyecta al cuerpo enfermo como objeto de conocimiento en la delirante tensión entre lo normal y lo patológico. El cuerpo perturbado y alterado es visto como una anomalía de un estado de salud idealizado.

Este discurso médico experto se desarrolla y perfecciona en la clínica durante la segunda mitad del siglo XIX, aunque como fruto de un proceso cuya genealogía deriva de la anatomía renacentista y que tiene su lugar de enunciación en la práctica médica colombiana y española. La construcción de este campo epistemológico estuvo articulado por dos dominios imbricados, el hospitalario y el pedagógico; el primero de los cuales es el espacio de positivización clínica donde el hecho patológico se clasifica como acontecimiento singular en la rejilla de comprensión médica de la época. El campo epistemológico de la racionalidad médica clasificatoria del siglo XIX tiene que ver con la preocupación por asimilar el acontecimiento patológico construido a partir de la relación médico/paciente, que se constituye como forma de conocimiento médico en el registro de la clínica, en tanto ciencia serial que busca frecuencias y homogeneidades de orden, para permitir comparaciones y extraer las identidades patológicas del caos de las singularidades. Se trata de una racionalidad clasificatoria (taxonomía y nosología) de un pensamiento de la vida que, para el caso de la medicina, entraña el catálogo de enfermedades donde se exponen las experiencias sensibles del dolor, las cuales dejan sus huellas como signos en el horizonte de comprensión dermatológica. Así, en la medicina, la nosología es el tercer dominio de la ciencia de la clasificación, junto a la botánica y la zoología. En estos dominios la vida es una categoría de clasificación:

> La salud de los hombres, el mejoramiento de sus condiciones, estaba finalmente en juego en las discusiones académicas sobre las flores más lejanas o sobre sus semillas reducidas, y también en las numerosas monografías (de la curiosidad) sobre las jibias, cangrejos, gusanos de seda, murciélagos, ballenas, caracoles etc. ¿Por qué? Porque el triunfo de los naturalistas y de sus repertorios irían a extenderse a la teoría de las enfermedades, a animarla e imponerle un método, sugerirle reglas y categorías (Dagognet, 2001, p.5).

En este sentido, la enfermedad desconcierta por su naturaleza multiforme. Por ejemplo, la inflamación, según el órgano que toque, puede ser o un flemón (piel), una erisipela (dermis), un catarro (mucosa) o una flogosis y exantema. Este es precisamente el terreno de la clínica dermatológica ligada a una razón clasificatoria en el orden del ser vivo.[8] El acontecimiento patológico, como un hecho discursivo encarnado en el cuerpo, tiene toda su operatividad en la relación médico/paciente desde el registro de la clínica, desde el cual se intenta rostrificar las entidades patológicas al establecer frecuencias, recrear estructuras homogéneas de orden y hacer comparaciones para establecer un territorio de discursividad en la experiencia de lo patológico. El dominio hospitalario depende íntegramente del pedagógico. El registro cuidoso realizado por los médicos, una descripción densa del estado patológico, del trascurso de enfermedades como la sífilis, la lepra, el lupus, el cáncer, la tuberculosis o los tumores, sus recurrencias y la exposición analítica en la cama del paciente, hace que el pensamiento clínico se soporte como ciencia y pedagogía. Como dice Foucault (2001a, p.159), "se da a conocer al darse a reconocer", puesto que es solo lo que se constata como experiencia sensible y repetible, lo que es aprehendido como conocimiento clínico, donde la singularidad del sujeto enfermo existiría, en su materialidad discursiva, en la razón clasificatoria del saber-poder médico, lo cual permitió el paso del enfermo a la enfermedad en un proceso de desterritorialización entre síntoma y signo en la potencialidad de lo que pueden los cuerpos en sus circunstancias. Es la transmisión de un conocimiento médico en el registro de una pedagogía del hecho patológico, en el que el acto de conocer, dar a conocer y reconocer remite a un ejercicio de educación de la mirada en la mediación entre maestro y alumno (dominio hospitalario/dominio pedagógico).

Es menester recordar que el siglo XIX hará de los fenómenos vitales normales y los patológicos una identidad como dogma científico en el campo discursivo de la medicina. Esta relación operará en otros dominios como el filosófico y psicológico, a partir de los cuales biólogos y médicos cimentarán sus contenidos de saber científico. En Francia – cuya clínica estuvo a la orden del día en la Colombia y la España de la época[9] – fueron Auguste Comte (1798-1857) y Claude Bernard (1813-1878) quienes expusieron ese dogma. Comte pone en escena las teorías médicas de François-Joseph-Victor Broussais (1772-1838) y orienta la cuestión desde lo patológico con el fin de determinar las leyes de lo normal. Comte toma la enfermedad como recurso para entender los fenómenos de la vida: "La identidad de lo normal y de lo patológico es afirmada para beneficio del conocimiento de lo normal" (Canguilhem, 1971, p.21). Con Claude Bernard (1976) y su *Introducción al estudio de la medicina experimental* (primera edición de 1865) el interés se desplaza de lo normal a lo patológico para actuar racionalmente sobre lo patológico. "El conocimiento de la enfermedad es buscado a través de la fisiología, con el objeto de fundar una terapéutica que rompa con el empirismo" (Cardona Rodas, 2005, p.157). Esta práctica médica pone en juego el lenguaje clínico y recurre a las representaciones iconográficas de enfermedades de la piel para darle eficacia simbólica a las formas de ver y las maneras de decir las experiencias subjetivas del sufrimiento.

Al transformar el sufrimiento humano en conocimiento médico, el ojo que habla se vuelve el asistente de las cosas del maestro de la verdad,[10] recurriendo en muchas ocasiones a imágenes como estrategia comunicativa en el ejercicio de la pedagogía médica. Como lo enuncia Foucault (2001a, p.167): "Una mirada que escucha y una mirada que habla: la experiencia clínica representa un momento de equilibrio entre la palabra y el espectáculo … todo lo visible es enunciable y es íntegramente visible porque es íntegramente enunciable". El vínculo entre lo visible y lo enunciable de la mirada clínica tuvo su mayor refinamiento en el horizonte discursivo de la dermatología en el siglo XIX. El estudio de las enfermedades de la piel, dermatosis, permitió al saber clínico la configuración de un espacio tangible del cuerpo enfermo, al inventariar y caracterizar las manifestaciones de lo conceptualizado como patología, proyectando una experiencia clínica de sensibilidad concreta en las superficies patológicas. Aquí imagen y palabra cumplen una función didáctica en la transmisión del conocimiento médico, en el decir verdad sobre el estado patológico.

Abordar la imagen como una estrategia comunicativa de la pedagogía clínica hace pensar en el orden de los signos. Siguiendo al lógico norteamericano Charles Sanders Peirce (1839-1914), a partir de su libro *Fragmentos de la ciencia de la semiótica*, de 1978, se puede establecer una clasificación de los signos como indicio, ícono y símbolo en relación con el objeto designado:

> El indicio es un fragmento de un objeto o algo contiguo a él, parte de un todo. En este sentido, una reliquia es un indicio: el fémur del santo en un relicario es el santo. O la huella de paso en la arena, o el humo del fuego en la lejanía. El icono, por el contrario, se parece a la cosa, sin ser la cosa. El icono no es arbitrario sino que está motivado por una identidad de proporción o forma. Al santo se le reconoce a través de su retrato, pero ese retrato se añade al mundo de la santidad, no es dado con él. Es una obra. El símbolo, por su parte, no tiene ya relación analógica con la cosa sino simplemente convencional: arbitrario con relación a ella, el símbolo se descifra con ayuda de un código. Así, la palabra 'azul' en relación con el color azul (Debray, 2009, p.183; destaque en el original).

Esta clasificación de los signos proyecta procedimientos designativos que se compenetran en el acto enunciativo de representar la enfermedad por parte de los médicos colombianos y españoles de la segunda mitad del siglo XIX. Este sentido, cuando los médicos recurren a las imágenes de enfermedades ven en estas manifestaciones sígnicas donde operan los iconos, pues la imagen de una enfermedad determinada resulta ser un retrato de lo que es percibido como anormal, patológico, grotesco o que perturba un estado de salud idealizado. Así, una representación iconográfica de una enfermedad es un signo expresivo en cuanto al hecho de su producción en sí mismo que, siguiendo a Charles Morris (1971), está destinado a un intérprete quien ve en un signo, en este caso un ícono, algo que produce su representación que condensa, transfigura e interpreta. Aquí intérprete y productor no son sujetos sino inmanencias sígnicas de atribución de sentido en las formas simbólicas de representar el cuerpo bajo los rigores de una enfermedad. Todos somos sujetos y objetos del lenguaje (Millas, 2014).

Al fundamentar su conocimiento en la mirada, el dermatólogo configurará una imagen de la enfermedad a partir de la experiencia de sensibilidad concreta de sus pacientes, registrando recurrencias patológicas, divulgando su conocimiento en la comunidad médica a partir de la publicación de sus hallazgos en revistas, tesis o clases magistrales que tienen por objetivo la transmisión de un saber con soporte de verdad científica. Por esto, recurrirá a diversas formas para representar el cuerpo enfermo o contratará a dibujantes y fotógrafos. Una de las primeras técnicas icónicas de reproducción y comunicación de las lesiones patológicas, que pone en escena sensibilidades de y por la piel, fueron los modelados en cera de la dermatología clínica del Hospital de San Luis, inaugurada por Jean-Louis Alibert (1768-1837) a comienzos del siglo XIX. Una de esas imágenes se puede apreciar en la Figura 1 del caso de una dermatitis hipertiforme analizada en aquel hospital por los médicos Bernier y Jean-Alfred Fournier en 1895:

> Los modelados tienen cualidades únicas: tridimensionales, coloreados de manera idéntica, con un realismo perfecto. Son realmente impresionantes, restituyendo así, además de la objetiva dermatosis, el sufrimiento del enfermo y la emoción de quien lo mira. Ciertamente, el arte del modelador no tiene como objeto hacerlo bello, sino hacerlo cierto. Los modelados también tienen defectos: poco desplazables, voluminosos, difícilmente reproducibles, se prestan mal a la comunicación que es uno de los objetivos de la iconografía dermatológica (Wallach, 1995, p.95).

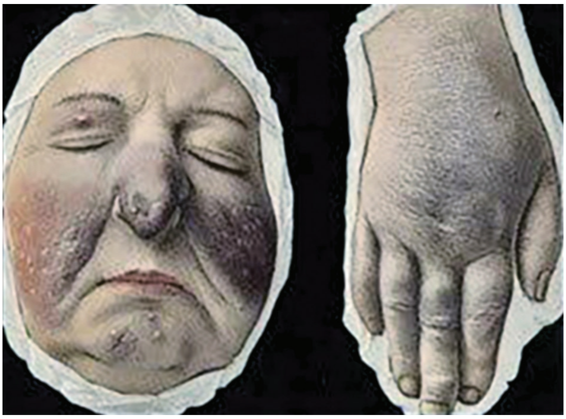

Figura 1: Dermatitis hipertiforme, vaciado en cera de l'Hôpital Saint-Louis de París, plancha n.2 (Besnier, Fournier, 1895-1897)

La puesta en escena de la iconografía dermatológica se refleja en la colección de representaciones de enfermedades de los médicos franceses Ernest Henri Besnier (1831-1909) y Jean-Alfred Fournier (1832-1914), publicada entre 1895 y 1897, donde se muestra una serie de moldeados en cera que "intentan capturar el sufrimiento del paciente y conmover la mirada de quien sitúa su atención en la representación" (Cardona Rodas, 2014, p.221). En tanto un espacio de interacción simbólica para la pedagogía médica, el uso de moldeados en cera de dermatosis se puso de manifiesto en la colección de enfermedades cutáneas y la sífilis de l'Hôpital Saint-Louis de París.[11] Los médicos en Europa y América implementaron las representaciones iconográficas en sus libros y estudios de enfermedades publicados en revistas médicas para darle eficacia discursiva a sus descripciones clínicas, sin olvidar las potencialidades icónicas de cada una de las observaciones de casos de enfermedades de la piel. Esta es una manera de transmisión del conocimiento médico, en la trama entre imagen y palabra, en la segunda mitad del siglo XIX.

Por las dificultades técnicas enumeradas por Wallach atrás, el lugar de los modelados en cera se lo apropiará la fotografía como técnica de visualización clínica y pedagógica más eficaz para el estudio de las enfermedades de la piel. En la puesta en escena de las fotografías (como acto performativo), "tres personajes encuentran su lugar: el paciente que expone sus padecimientos y que por lo regular no podía ser sanado; el médico que emprende la descripción dermatológica de la enfermedad y el fotógrafo, observador científico que pone su arte al servicio" (Cardona Rodas, 2014, p.222) de una reproducción de lo patológico ligada a la precisión descriptiva y la "e-moción" (en su dimensión intersubjetiva) del sufrimiento visible descrito por la práctica médica en sus descripciones patológicas, que en la iconografía dermatológica en siglo XIX tuvo toda su potencia enunciativa (Calcagno-Tristant, 2004; Tilles, 2011). La relación entre esos tres personajes y entre la descripción y la “e-moción” ante la enfermedad deformante se pone en juego, por ejemplo, en la serie de fotografías retocadas de enfermos de l’Hôpital Saint-Louis de París de los médicos Alfred Hardy (1811-1893) y Aime de Montméja (Hardy, Montméja, 1868), así como en las cincuenta fotografías retocadas con acuarela de Félix Méheux (1838-1908) que ilustraron el libro *Précis iconographique des maladies de la peau*, de E. Chatelain, publicado en París en 1896.

El estudio del hecho patológico y su materialidad sensible en la pluralidad de sus manifestaciones autorizó a la medicina convertir el sufrimiento en conocimiento al plantear una tensión problemática entre lo visible y lo decible. La función de la imagen y de la descripción médica se despliega entre la instrucción, el espectáculo y el conocimiento por redundancia visual y narrativa (sensibilidad concreta y semiología clínica). Según Ricardo Guixà Frutos (2012, p.53-73), el dispositivo fotográfico en el siglo XIX permitió la puesta en imagen de una política de la representación del otro y de lo otro, marcando de forma indeleble el imaginario colectivo en relación con el concepto de alteridad. En el caso de disciplinas como la antropología o saberes como la medicina, se implementará la imagen fotográfica para reportar la existencia de lo extraño, lo foráneo, es decir, todo aquello que se encontrara por fuera del campo irradiante civilizatorio europeo. Pero, la representación del otro inquietará la presencia de lo mismo, pues al retratar al bárbaro como objeto de estudio este ya no estará solo en las antípodas, sino también en los cuerpos deformados por los avatares de enfermedades como la sífilis y la lepra. Estos cuerpos también serán fotografiados y retocados en una política de la exhibición de lo monstruoso y lo deforme, representando un devenir otro por mutación somática de lo mismo.

> La fisionomía, la antropología y algunas ramas de la medicina [la clínica dermatológica] encontraron en la cámara la herramienta para sus pesquisas. El retrato se volvió ciencia por mediación de la fotografía, adquiriendo esta especial categoría de conocimiento gracias a la fidelidad y precisión atribuidas a los documentos por ella suministrados (Guixà Frutos, 2012, p.55).

Unido a una retórica de la imagen fotográfica, la diagnosis de enfermedades, desde una perspectiva anatomo clínica, a finales del siglo XIX, pone en juego una relación con el análisis diferencial de las distintas manifestaciones patológicas que tienen como escenario la piel. Las revelaciones cutáneas o superficies patológicas registradas por la dermatología clínica le dan materialidad a la enfermedad, al ser un territorio de visibilidad y decibilidad de lo patológico. Por esta razón, toda enfermedad será comprendida desde un campo de materialidad dermatológica.

## El uso de la imagen médica para una pedagogía clínica del cuerpo enfermo

Durante los siglos XIX y XX, en cuanto a las relaciones entre modelo biomédico y uso de imágenes en las revelaciones del cuerpo anatómico (Sirvent Ribalda, 2013), se mantendrá en medicina el uso de los dibujos, vaciados y moldes en cera desarrollados en el XVIII, como la nueva tecnología que significó la fotografía retocada,[12] para representar los casos clínicos de enfermedades. En el contexto de la biomedicina, el uso de imágenes es un intento de mejorar la comprensión del cuerpo anatómico en el,

> … fenómeno de re-significación del cuerpo humano en las ciencias médicas, y a la vez de contextualizar la articulación entre las nociones de cuerpo y la que posee el modelo médico, la mirada ha sido antropológica. Esta mirada ha servido para captar el campo de significación de las formas de pensar y de actuar que conlleva la (re) significación del cuerpo humano y la relación entre Medicina y la Antropología médica, especialmente el modelo crítico abre la puerta a una mirada antropológica de la Medicina a partir de ensayos, etnografías y biografías (más o menos críticas con el modelo) de los protagonistas, médicos o enfermeros sobre sus propias experiencias (Sirvent Ribalda, 2013, p.26).

Los atlas de dermatología clínica del siglo XIX, donde se consignaban fotografías de casos de enfermedades de la piel, implementaban la técnica del iluminado o coloreado a mano. Esta técnica consistía en añadir manualmente color a una fotografía en blanco y negro con el objetivo de aumentar el realismo, darle una eficacia visual a la descripción y ubicación de la enfermedad, además de tener propósitos artísticos. Los materiales implementados eran acuarelas, óleos, lápices de colores o pasteles, pinturas y tintes aplicados sobre la superficie de la imagen por medio de pinceles, dedos y bastoncillos de algodón. En la actualidad, se utilizan aerógrafos. Las fotografías coloreadas a mano fueron muy populares a finales del siglo XIX, antes de la invención de la fotografía a color, llegando a existir empresas especializadas en su producción. Después de la invención del daguerrotipo, en 1839, esta técnica tuvo su apogeo. El primer daguerrotipo coloreado a mano corresponde al pintor y grabador suizo Johann Baptist Isenring (1796-1860), quien utilizó, en 1840, una mezcla de goma árabe y pigmentos en daguerrotipos. También hubo otros esfuerzos para producir imágenes fotográficas de color. Daniel Davis Jr., en 1842, patentó un método para colorear daguerrotipos por medio de galvanoplastia, método que sería mejorado al año siguiente por Warren Thompson. Este método fue pronto abandonado. La técnica de iluminado o coloreado a mano se mantuvo hasta la mitad del siglo XX, cuando la Eastman Kodak Company presentó la película de color Kodachrome.

En este sentido, su ubica el trabajo de fotografía médica de los mencionados Alfred Hardy y Aimé de Montméja, quienes configuraron una práctica médica iconográfica de exhibición del cuerpo enfermo en la serie de fotografías de pacientes del Hôpital de Saint Louis en París de 1868, hospital donde ambos fundaron el primer servicio fotográfico dedicado a la pedagogía clínica. Lo llamativo de las fotos de este álbum se relaciona con la forma de presentar los fotogramas: Hardy clasificaba las enfermedades, mientras que Montméja toma las fotografías y las decora con técnica colorista para resaltar el paso de la enfermedad sobre el cuerpo retratado del paciente. Veamos dos de los fotogramas que integran la obra de Hardy y Montméja (Figuras 2 y 3) y una de las fotografías retocadas con acuarela de Félix Méheux publicada en el libro de Chatelain (Figura 4), donde están implicados tres personajes, como mencionábamos atrás (paciente, médico y observador científico) en el tejido entre la descripción médica y la “e-moción” del sufrimiento visible.

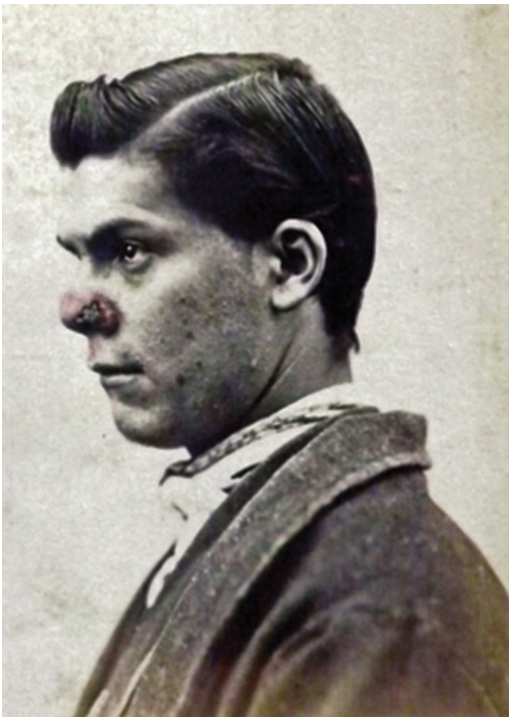

Figura 2: Scrofulide pustuleuse. Fotografía de la colección de l’Hôpital Saint-Louis de París, retocada con técnica colorista (Hardy, Montméja, 1868)

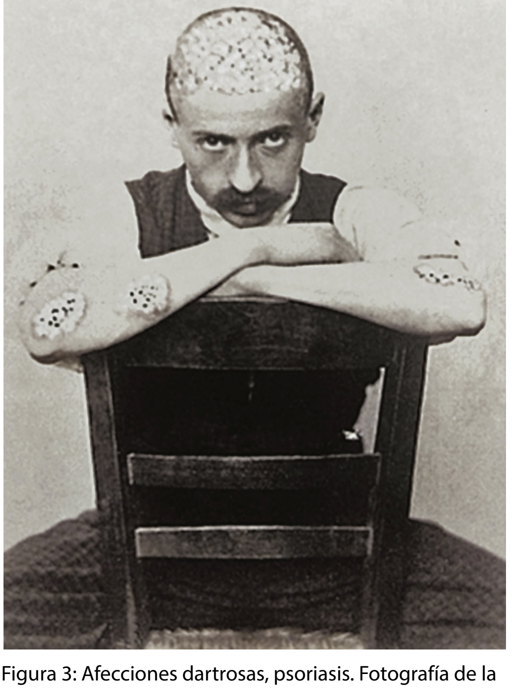

Figura 3: Afecciones dartrosas, psoriasis. Fotografía de la colección de l’Hôpital Saint-Louis de París, retocada con técnica colorista (Hardy, Montméja, 1868)

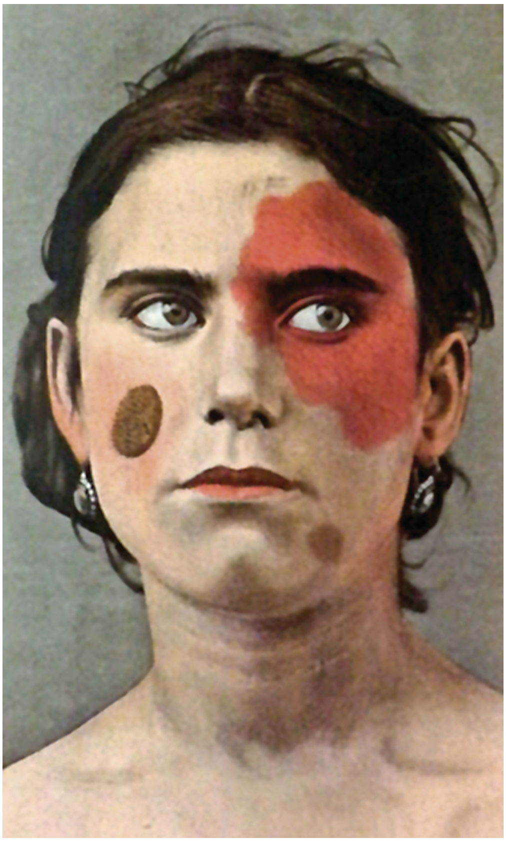

Figura 4: Noevi. *Précis iconographique des maladies de la peau*, plancha XXXIII. Fotografía retocada con acuarela, de Félix Méheux (Chatelain, 1896)

Las tres reproducciones indican el carácter performativo de la enfermedad, donde el cuerpo enfermo enseña las lesiones patológicas y las huellas de la alteridad de aquello que es percibido como repugnante. Las imágenes muestran, verbo que en su etimología se relaciona con monstruo, el rostro de la enfermedad en el orden de una perplejidad teatralizada, donde se conjugan imagen, tecnología, saber, gestos, enfermedad, cuerpos, miradas, dolor y puestas en escena para darle sentido a un dispositivo fotográfico que funciona como una máquina para hacer ver y para hacer hablar en la práctica discursiva de la dermatología desde su preocupación pedagógica. Así, las imágenes del archivo visual médico narran las experiencias del dolor, la pretensión de objetividad del conocimiento médico y la captación de lo real mediado por el dispositivo fotográfico o la intensión comunicativa de la imagen que entreteje cuerpo, acontecimiento y lenguaje. "La emoción, el dolor, la angustia, los afectos que produce la imagen, no quedarán excluidos del conocimiento que la imagen fulgurante y relampagueante ofrece, destello que no nos deja más que su propia retirada y alguna huella de su impacto…" (Fisgativa Sabogal, 2013, p.170), puesto que la imagen quema por su campo de inmanencia con lo real, el deseo, la destrucción, el brillo, el movimiento, el dolor y la memoria (Didi-Huberman, 2008).[13]

Las representaciones iconográficas de enfermedades de la piel reflejan la inquietud epistemológica propia de la medicina decimonónica, que intenta darle un espacio de legibilidad a toda descripción clínica de enfermedades, en términos de una tensión problemática entre lo que se ve y lo que se dice, al convertir en conocimiento médico los espacios sensibles de la experiencia subjetiva de la enfermedad, según una operación perceptiva propia de la clínica que hace de dicha experiencia un hecho patológico. El propósito de la imagen, en la diversidad de sus manifestaciones, junto con las descripciones médicas de los pacientes, gravita entre el conocimiento por insistencia, la instrucción y el espectáculo ante una estética del cuerpo alterado, donde lo visual y lo narrativo son la base de una territorialidad de sensibilidad discursiva de la experiencia clínica. Con todo esto, la descripción clínica de enfermedades deformantes pone de manifiesto un saber en acción pedagógica del campo de la dermatología en la segunda mitad del siglo XIX, según una analítica para la compresión de las diversas experiencias morfológicas, producto de los recorridos patológicos que dejan huella en el cuerpo enfermo. Una dramaturgia epidérmica se desata en el conocimiento médico en términos de un proceso de rostrificación del estado de enfermedad, al hacerlo inteligible por la mirada clínica dermatológica. De esta forma, "producir en el pensamiento clínico decimonónico remite a un dejar huella en la piel, tatuarla e inscribirla en superficies de topografías expresivas: piel historiada, lugar de memoria y de rugosidades existenciales" (Cardona Rodas, 2014, p.223).

Con el objetivo de afianzar lo que aquí se entiende por representación, recordemos que el prefijo "re" hace referencia al sentido de una sustitución y el sufijo "presentar" se inscribe como una manifestación de intensidad. Así, las formas simbólicas de la "re-presentación" de la enfermedad están dadas por la construcción en contenido y expresión, en el plano de la palabra y la imagen, de un artificio que manifiesta tanto una sustitución como una intensidad, es decir, un acontecimiento enunciativo que intenta capturar al cuerpo bajo los rigores de la enfermedad. Por esta razón, sostiene Michel Serres (2002, p.26) que

> … la piel historiada lleva y muestra la vida propia o la visible: desgastes, cicatrices causadas por las heridas, porciones de piel entumecidas por el trabajo, arrugas y surcos de antiguas esperanzas, manchas, lunares, eczemas, psoriasis, paños, allí se imprime la memoria, por qué buscarla en otra parte; o la invisible: huellas fluctuantes de las caricias, recuerdos de la seda, de la lana, los terciopelos, las pieles, los fragmentos de roca, las cortezas rugosas, las superficies rasposas, los cristales de hielo, las llamas del fuego, timideces del tacto sutil, audacias del contacto combativo. A un dibujo o colorido abstracto correspondería un tatuaje fiel y leal donde lo sensible se expresaría: si este imita viñetas, iconos o letras, todo se hundiría en lo social. La piel se convertiría en bandera, en la medida que presenta huellas.

En esta lógica de la "re-presentación", el ojo captura una resonancia entre la voz de la enfermedad como experiencia sensible y la mano que configura una escritura de la enfermedad, pues aquello que se rostrifica como patológico constituye una escritura del saber de la enfermedad guiado por las inscripturas estudiadas en sus recurrencias en los cuerpos de los pacientes. Las Figuras 2, 3 y 4 muestran este saber de la enfermedad proyectado en una relación entre fotografía retocada y descripción clínica, en aquella dramática epidérmica de las experiencias corporizadas de lo patológico que le da material reflexivo a la medicina de la época. La piel aquí es la superficie de inscripciones múltiples de la enfermedad, de devenires-intensos entre lo que se ve y lo que se dice en el registro de la dermatología clínica en la segunda mitad del siglo XIX. Una fotografía, manipulada por la técnica de iluminado o coloreado a mano, sería una evidencia en la trama epistemológica entre imagen y palabra en el ejercicio del conocimiento médico.

La imagen capta lo real en un proceso de remisión simbólica frente a lo que se ve y aquello que puede ser visto. Susan Sontag (2006, p.216) sostiene que la imagen podría usurpar la realidad en el caso de una fotografía. Según la autora, no es

> … solo una imagen (en el sentido en que lo es una pintura), una interpretación de lo real; tampoco es un vestigio, un rastro directo de lo real, como una huella o una máscara mortuoria. Si bien un cuadro, aunque cumpla con las pautas fotográficas de semejanza, nunca es más que el enunciado de una interpretación, una fotografía nunca es menos que el registro de una emanación (ondas de luz reflejadas por objetos), un vestigio material del tema imposible para todo cuadro.

## Consideraciones finales

A través de un ejemplo iconográfico sería posible, según el orden del discurso de una arqueología de lo visual en el campo del conocimiento médico, sintetizar lo que se ha venido argumentado en este artículo. Se trata de la fotografía de un caso de lepra en una niña, publicada en 1868 en su álbum por Hardy y Montméja. En esta fotografía (Figura 5), como en todas las que componen el álbum, se proyecta el complejo de fuerzas que subyacen en la dermatología clínica, la cual intenta capturar las experiencias sensibles de lo patológico al representarlas como íconos y al figurarlas como lenguaje. Con esta fotografía es perceptible una retórica de la imagen médica en la rostrificación maquínica semiótica basada en la distinción positivista entre lo normal y lo patológico y que, a la vez, intenta capturar lo inclasificable al mezclar lo imposible y lo prohibido ante lo que causa horror al perturbar una norma anatomo fisiológica.

Lo que se nombra se ve como lenguaje y lo que sorprende se clasifica como repulsivo, experiencias desnudas del orden de lo que puede un cuerpo en sus circunstancias. El saber de la enfermedad de la dermatología clínica, de la segunda mitad del siglo XIX, le da espacio de visibilidad a lo que explora ante un cuerpo que experimenta los trazos de la enfermedad, huellas que son analizadas en el espesor de una geometría del cuerpo alterado y captado en la imagen médica (dibujo, vaciados en cera o fotografía retocada) y desplegada en las narrativas corporizadas del pensamiento clínico. La niña de la Figura 5 exhibe las huellas de la enfermedad en su cuerpo en la materialización de una experiencia del dolor, su mirada inclinada, sus dedos entrecruzados y su piel deformada contrastan con los pliegues de su vestido, el cual resalta el recorrido trágico y agónico que la fotografía intenta proyectar. La enfermedad se manifiesta con hipertrofias que desgarran la estructura normal de su cuerpo, descomponen su piel ante la mirada inmutable del ojo que capta su sufrimiento. Esto no es la enfermedad sino su (re)presentación, en su grafía performática, ante lo que se intensifica, redobla y se muestra en una sociedad que ve el signo de la podredumbre en el retrato de esta niña. La imagen proyecta sus llamas ante el despliegue de lo deforme escenificado en las experiencias sensibles de la alteridad. La niña contempla su corrupción. Sin embargo, no es la que se degrada, sino el ojo que ve en ella una degradación.

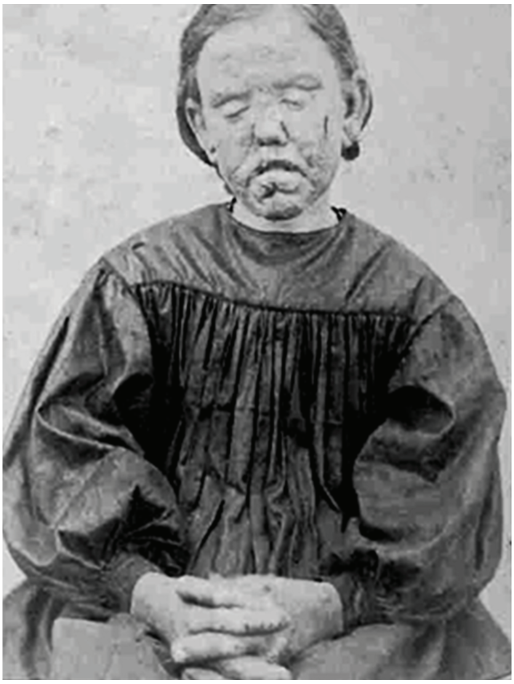

Figura 5: Lepra. Fotografía de la colección de l'Hôpital Saint-Louis de París (Hardy, Montméja, 1868)

Esta fotografía permite volver al inicio de este artículo, pues con el poema *Venus anadiomena* de Rimbaud se ve el ascenso de un cuerpo transfigurado, resultante de la contemplación de sí mismo como hojaldre al ofrecer la representación inversa que espanta como reflejo de lo mismo y lo otro, que aparece en la misma imagen donde un cuerpo es calcinado por el paso de la sífilis, enfermedad que evoca ese miedo colectivo de ser tocado por una corporeidad degradada. No se ve, entonces, un caso de lepra o una mujer que se descompone por la sífilis, sino que, por sustitución e intensidad en la imagen que proyecta la fotografía y el poema, se ve un campo de sensaciones, repudios, odios y temores que una sociedad tiene de sí misma en la rostrificación de lo patológico. Por esto, la imagen pone en juego la presencia en ausencia de una memoria condensada que yace como acontecimiento patológico y que, para el caso del poema de Rimbaud, se agita en un cuerpo que se degrada ante el paso de una úlcera tebrosa, lo cual tiene su materialidad afectante en los repertorios corporales visuales de enfermedades que fueron consultados en los archivos médicos en Colombia y España.

El pensamiento clínico dermatológico de la época organiza un espacio de visibilidad que pretende explorar lo que tiene delante, e informa a los sentidos en una geometría que da volumen a la percepción de lo que es medido como patológico. La bruma de lo que está oculto es aclarada por una lectura rigurosa de la mirada que toca, oye y ve, proyectando un lenguaje abrasador.

## NOTAS

* Este artículo es resultado de la investigación doctoral titulada *Iconografías médicas: dermatología clínica en Colombia y España durante la segunda mitad del siglo XIX*, presentada en 2016, en la Universitat Rovira i Virgili, de Tarragona, España (disponible en: https://www.tdx.cat/handle/10803/399229?locale-attribute=ca_ES#page=1).

[1] El ideal de normalidad física y psicológica se aprecia en la necesidad de coleccionar especímenes singulares en la museología teratológica decimonónica en las facultades de medicina. La colección de *History of anthropology* de la University of Wisconsin Press posee referencias al respecto, como por ejemplo *Objects and others: essays on museums and material culture*. Ver George W. Stocking (1996).

[2] A este respecto, Gilles Deleuze (1989, p.14), en su lectura de la obra filosófica de Leibniz, a partir del concepto de pliegue, sostiene que "un cuerpo flexible o elástico todavía tiene partes coherentes que forman un pliegue, de modo que no se separan en partes de partes, sino que más bien se dividen hasta el infinito en pliegues cada vez más pequeños que conservan siempre una cierta cohesión". Esta cohesión se halla en las superficies patológicas analizadas por la práctica médica desde la rejilla de comprensión clínica que capta-describe la enfermedad ante los rigores de lo que puede un cuerpo.

[3] La expresión "lo que puede un cuerpo" corresponde a una crítica a la filosofía mecanicista cartesiana que pone en juego Baruj Spinoza en su obra *Ética demostrada en orden geométrico*, escrita en 1661 y publicada en 1675, donde cuestiona la distancia entre cuerpo y alma (*res extensa* y *res cogitans*) que fundamenta la duda metódica de Descartes y que refleja los dualismos de la tradición occidental. Según Spinoza (1980, p.127): "Y el hecho es que nadie, hasta ahora, ha determinado lo que puede el cuerpo, es decir, a nadie ha enseñado la experiencia, hasta ahora, qué es lo que puede hacer el cuerpo en virtud de las solas leyes de su naturaleza, considerada como puramente corpórea, y qué es lo que no puede hacer salvo que el alma lo determine. Pues nadie hasta ahora ha conocido la fábrica del cuerpo de un modo lo suficientemente preciso como para poder explicar todas sus funciones, por no hablar ahora de que en los animales se observan muchas cosas que exceden con largueza la humana sagacidad, y de que los sonámbulos hacen en sueños muchísimas cosas que no osarían hacer despiertos; ello basta para mostrar que el cuerpo, en virtud de las solas leyes de su naturaleza, puede hacer muchas cosas que resultan asombrosas a su propia alma. Además, nadie sabe de qué modo, ni con qué medios, el alma mueve al cuerpo, ni cuántos grados de movimiento puede imprimirle, ni con qué rapidez puede moverlo. De donde se sigue que cuando los hombres dicen que tal o cual acción del cuerpo proviene del alma, por tener esta imperio sobre el cuerpo, no saben lo que se dicen, y no hacen sino confesar, con palabras especiosas, su ignorancia". Este punto de vista tiene su importancia para reflexionar sobre el cuerpo en términos de una experiencia intersubjetiva de aquello que afecta y es afectado ante los rigores de la enfermedad deformante, que en el horizonte de comprensión en este artículo se ubica en los registros analizados en archivos médicos de la segunda mitad del siglo XIX en Colombia y España.

[4] El campo de investigación sobre la historia de la fotografía médica en América Latina ha tenido un espectro de reflexión heteróclita. Trabajos como los de Cueto (1999); Ciancio y Gabriele (2012); Ciancio (2009); Pantoja (2022); Cuarterolo (2009); Guixà Frutos (2012); Cardona Rodas (2011, 2014, 2016); Gorbach (2020, 2013, 2008); y Cardona Rodas y Vasquez Valencia (2011), para solo mencionar algunos, han mostrado cómo el uso del dispositivo de la imagen en las formas de ver y nombrar el cuerpo enfermo está en directa relación con una ilusión de objetividad científica propia del positivismo médico del siglo XIX.

[5] El uso de la fotografía hace parte de las estrategias argumentativas y expositivas que el saber médico puso en juego para la producción de conocimiento científico de la enfermedad en la cultura visual e impresa que circuló en la prensa en la segunda mitad del siglo XIX en Europa y América, lo cual analizó Maria Claudia Pantoja (2022) en su artículo sobre medicina y cultura visual en la difusión del conocimiento médico experimental en Argentina entre 1890 y 1915. Así, "la técnica fotográfica, que permitía aislar, encuadrar y repetir casos similares en un formato transportable, fue fundamental para la estandarización y la multiplicación de las observaciones en distintos momentos y por parte de diferentes actores. Con una cámara era posible tomar imágenes de un modo relativamente sencillo de varios sujetos que padecían dolencias semejantes; también de una misma persona desde diversos ángulos o en diferentes momentos para registrar la progresión de una patología o de su proceso de cura" (p.710).

[6] Joan Fontcuberta (2011) sostiene que existe un realismo ilusorio en la toma fotográfica encarnado en una ortopedia de la apariencia. "La historia de la fotografía puede ser contemplada como un diálogo entre la voluntad de acercarnos a lo real y las dificultades para hacerlo. Por esto, a pesar de las apariencias, el dominio de la fotografía se sitúa más propiamente en el campo de la ontología que en el de la estética" (p.13). Aquí se situa el problema del *punctum*, explorado por Roland Barthes (2010), en una fotografía que, para el caso de la imagen médica, proyecta una serie de valores y ritmos propios de un saber permeado por percepciones de lo horrendo, lo deforme y lo degradado en función de la imagen que se tiene de lo normal, lo cual no está contenido originariamente en la fotografía al ser un dispositivo de representación.

[7] Estos médicos figuran en el repertorio de personalidades que los médicos colombianos y españoles decimonónicos implementan en sus caracterizaciones clínicas de las "enfermedades cutáneas", generando así un discurso sabio en torno de la configuración de un campo de saber médico ligado a la ilusión de objetividad inherente al positivismo.

[8] Este campo de reflexión se desarrolla en Coleman (1983); Dagognet (2001); Cardona Rodas, (2011, p.171-202, 2012); Foucault (2001a; 2001b); Castro-Gómez (2010); Gerbi (1960); Jacob (1988), Jay Gould (1997); Johannisson (2006); Todorov (2000); Tort (1989) y Vigarello (2006).

[9] Esta transmisión del saber médico en la segunda mitad del siglo XIX, desde sus ritornelos discursivos (Deleuze, Guattari, 2004, p.317-358) singulares en la comprensión de la experiencia sensible de la enfermedad como lenguaje corpoafectante, es uno de los temas de la tesis doctoral en antropología denominada *Iconografías médicas: dermatología clínica en Colombia y España en la segunda mitad del siglo XIX* (Cardona Rodas, 2016).

[10] El maestro de la verdad refiere al problema de la verdad, *aletheia* en griego, como un juego de posiciones de sujeto en función de la palabra que puede ser dicha en un sistema de pensamiento que responde a su vez un sistema social. Marcel Detienne (1983) examina que el problema de la verdad, como un ejercicio de la palabra, se instala en el pensamiento griego arcaico, donde se hace de ella algo objetivo y racional que se distancia, sin dejarla atrás, de la explicación mítica. La racionalidad occidental ligada a la demostración, verificación y experimentación encuentra su actuación en la práctica médica que hace del conocimiento una operación discursiva de distinción, en cuanto a la posición de sujeto dicente del médico al decir verdad sobre el estado patológico.

[11] Estas colecciones que figuran también en las facultades de medicina en Colombia y España en la segunda mitad del siglo XIX, la cuales tenían por objetivo la educación de la mirada clínica, sin dejar de advertir su valor en una historia del arte desde el campo de la medicina.

[12] La interpretación de las superficies patológicas, como hemos visto, están presentes en los libros de los dermatólogos franceses Ernest Besnier (1831-1909) y Jean-Alfred. Fournier (1832-1914), como el mencionado de Hardy y Montméja. Entre 1900 y 1904, junto con Louis-Anne-Jean Brocq (1856-1928) y Lucien Jacquet (1860-1914), Besnier publicará una serie de tomos sobre la práctica dermatológica donde utiliza tanto los vaciados en cera como la imagen fotográfica para darle cuerpo enunciativo a las caracterizaciones de enfermedades de la piel. La tesis de Auguste-Joseph-Marius Burais sobre la aplicación de la fotografía en medicina constituye un texto importante para comprender la técnica fotográfica y las posibilidades que brindaba este dispositivo tecnológico en la segunda mitad del siglo XIX para darle un soporte visual a las caracterizaciones de enfermedades, entre ellas las de la piel. Ver Burais (1896); Besnier y Fournier (1895-1897); y Besnier, Brocq y Jacquet (1900-1904). Estos referentes bibliográficos sobre la historia de la enseñanza de la dermatología en la segunda mitad del siglo XIX hacían parte del material de estudio de las Facultades de Medicina en Colombia y España.

[13] Estos problemas que convocan a una reflexión sobre una arqueología del poder de las imágenes están presentes igualmente en los trabajos de Belting (2012); Didi-Huberman (2009, 2020); Moxey (2016); Fontcuberta (2011); Freedberg (2018); y Mitchell (2017).
